# Deep learning-based quantification of brain atrophy using 2D T1-weighted MRI for Alzheimer’s disease classification

**DOI:** 10.3389/fnagi.2024.1423515

**Published:** 2024-08-14

**Authors:** Chae Jung Park, Yu Hyun Park, Kichang Kwak, Soohwan Choi, Hee Jin Kim, Duk L. Na, Sang Won Seo, Min Young Chun

**Affiliations:** ^1^Research Institute, National Cancer Center, Goyang, Republic of Korea; ^2^Department of Neurology, Samsung Medical Center, Sungkyunkwan University School of Medicine, Seoul, Republic of Korea; ^3^Neuroscience Center, Samsung Medical Center, Seoul, Republic of Korea; ^4^Alzheimer’s Disease Convergence Research Center, Samsung Medical Center, Seoul, Republic of Korea; ^5^BeauBrain Healthcare, Inc., Seoul, Republic of Korea; ^6^Department of Health Sciences and Technology, SAIHST, Sungkyunkwan University, Seoul, Republic of Korea; ^7^Department of Digital Health, SAIHST, Sungkyunkwan University, Seoul, Republic of Korea; ^8^Department of Neurology, Yonsei University College of Medicine, Seoul, Republic of Korea; ^9^Department of Neurology, Yongin Severance Hospital, Yonsei University Health System, Yongin, Republic of Korea

**Keywords:** brain atrophy, deep learning, 2D and 3D T1-weighted MRI, CSF volume, dementia, Alzheimer’s disease

## Abstract

**Background:**

Determining brain atrophy is crucial for the diagnosis of neurodegenerative diseases. Despite detailed brain atrophy assessments using three-dimensional (3D) T1-weighted magnetic resonance imaging, their practical utility is limited by cost and time. This study introduces deep learning algorithms for quantifying brain atrophy using a more accessible two-dimensional (2D) T1, aiming to achieve cost-effective differentiation of dementia of the Alzheimer’s type (DAT) from cognitively unimpaired (CU), while maintaining or exceeding the performance obtained with T1-3D individuals and to accurately predict AD-specific atrophy similarity and atrophic changes [W-scores and Brain Age Index (BAI)].

**Methods:**

Involving 924 participants (478 CU and 446 DAT), our deep learning models were trained on cerebrospinal fluid (CSF) volumes from 2D T1 images and compared with 3D T1 images. The performance of the models in differentiating DAT from CU was assessed using receiver operating characteristic analysis. Pearson’s correlation analyses were used to evaluate the relations between 3D T1 and 2D T1 measurements of cortical thickness and CSF volumes, AD-specific atrophy similarity, W-scores, and BAIs.

**Results:**

Our deep learning models demonstrated strong correlations between 2D and 3D T1-derived CSF volumes, with correlation coefficients *r* ranging from 0.805 to 0.971. The algorithms based on 2D T1 accurately distinguished DAT from CU with high accuracy (area under the curve values of 0.873), which were comparable to those of algorithms based on 3D T1. Algorithms based on 2D T1 image-derived CSF volumes showed high correlations in AD-specific atrophy similarity (*r* = 0.915), W-scores for brain atrophy (0.732 ≤ *r* ≤ 0.976), and BAIs (*r* = 0.821) compared with those based on 3D T1 images.

**Conclusion:**

Deep learning-based analysis of 2D T1 images is a feasible and accurate alternative for assessing brain atrophy, offering diagnostic precision comparable to that of 3D T1 imaging. This approach offers the advantage of the availability of T1-2D imaging, as well as reduced time and cost, while maintaining diagnostic precision comparable to T1-3D.

## Introduction

Neurodegenerative processes characterized by brain atrophy represent the final common pathway observed in most types of dementia, including Alzheimer’s disease (AD), frontotemporal dementia, and dementia with Lewy bodies (Rosen et al., 2002; Whitwell et al., 2007). Brain atrophy is a crucial biomarker that displays distinct patterns specific to each type of dementia (Young et al., 2020). Furthermore, the extent of brain atrophy is highly correlated with cognitive performance and is recognized as a predictor of future cognitive decline (Sluimer et al., 2008; Ikram et al., 2010).

Traditionally, for assessing brain atrophy, cortical thickness measurement and volumetric analysis have served as research surrogates (Lemaitre et al., 2012; Pini et al., 2016). These surrogate markers can be quantified using three-dimensional (3D) T1-weighted images from magnetic resonance imaging (MRI), offering improved diagnostic performance for research purposes. Despite its high diagnostic performance, the practical application of 3D T1 imaging in clinical settings is impeded by its time-consuming and costly acquisition process, thus limiting its clinical readiness. By contrast, clinical practice predominantly utilizes two-dimensional (2D) T1-weighted images from MRI images. Within these settings, radiologists and clinicians assess brain atrophy through visual examination, focusing on indicators, such as enlargement of the lateral ventricles (LVs), sulcal widening between the gyri, and the width of the temporal horn adjacent to the hippocampus (Koedam et al., 2011; Harper et al., 2015). Cerebrospinal fluid (CSF) volume, in particular, has been shown to correlate with brain atrophy, providing a valuable biomarker for neurodegenerative diseases (De Vis et al., 2016). However, these visual assessments tend to be less accurate and less precise than quantitative analyses, underscoring the need for accessible and quantitative methods based on 2D T1 images in clinical practice.

Recent advancements in deep learning have led to a few attempts to use 2D T1 images to predict brain atrophy (Marwa et al., 2023; Zhou et al., 2023), which is traditionally quantified via 3D T1 images. The Convolutional Neural Network is designed with an architecture that drew inspiration from the human visual cortex, mirroring the interconnectedness observed among neurons (Krizhevsky et al., 2012). Fully Convolutional Networks (Long et al., 2015) have found extensive application in semantic segmentation within the domain of computer vision. Through the application of deep learning, 2D T1 images with better clinical readiness may be reconstructed to quantify brain atrophy with a level of diagnostic accuracy approaching that of 3D T1 images.

A clinical decision support system (CDSS) enhances health-related decisions by integrating pertinent clinical knowledge and patient information, thereby improving healthcare delivery (Jerry Osheroff et al., 2012). In particular, non-knowledge-based CDSS make decisions using techniques, such as artificial intelligence, machine learning, or deep learning, rather than directly adapting the knowledge of medical experts (Sutton et al., 2020). Thus, the CDSS may contribute to filling the gap in unmet needs in clinical practice. In memory clinics, clinicians often encounter complex inquiries from patients, such as comparisons of their brains to dementia or age-related brain atrophy. To answer these questions, researchers have attempted to develop algorithms predicting the AD brain similarity score (Lee et al., 2018a) or brain age index (BAI) (Kang et al., 2023) using 3D T1 images. However, considering the practical limitations of 3D T1 images, algorithms based on 2D T1 images should be introduced in clinical settings.

In this study, we developed an algorithm that quantifies brain atrophy by measuring CSF volumes in the regions of interest (ROIs) including anterior and posterior lateral ventricles (LVs), sulcal widenings between the gyri in the frontal, temporal, parietal and occipital lobes, and the width of the temporal horn adjacent to the hippocampus using 2D T1 images. We also validated the clinical utility of this algorithm in terms of the differentiating patients with dementia of the Alzheimer’s type (DAT) from cognitively unimpaired (CU) individuals, prediction of AD-specific atrophy similarity, and calculation of atrophic changes (W-score) and BAI relative to age and sex, based on CSF measurements in the ROIs. Given that 2D T1 images are more commonly used in clinical practice than 3D T1 images, our practical approach may enable earlier diagnosis, timely treatment adjustments, and effective monitoring of disease progression.

## Materials and methods

### Participants

To develop our algorithm, 1,120 participants aged 55–90 years were recruited from the Alzheimer’s disease convergence research center at Samsung Medical Center (SMC) in South Korea (Supplementary Figure S1). All participants underwent neuropsychological tests, brain MRI (including 3D T1 images), and *APOE* genotyping. CU individuals had no objective cognitive impairment observed after a comprehensive neuropsychological test on any cognitive domain (above the-1.0-standard deviation [SD] of age-and education-matched norms in memory and below-1.5 SD in other cognitive domains) (Ahn et al., 2010). Participants with DAT met the diagnostic criteria of the 2011 National Institute on Aging and Alzheimer’s Association (McKhann et al., 2011). To calculate the W-score using an independent cohort, we included an additional 109 CU participants from the SMC.

We excluded participants who had any of the following conditions: (1) white matter hyperintensities due to radiation injury, multiple sclerosis, vasculitis, leukodystrophy or metabolic disorders; (2) traumatic brain injury; (3) territorial infarction; (4) brain tumor; and (5) rapidly progressive dementia.

The study protocol received approval from the Institutional Review Board of SMC, and all procedures were conducted in accordance with the approved guidelines. Written consent was obtained from each participant prior to their involvement in the study.

### Acquisition and preprocessing of 3D and 2D T1 images

A 3.0 T MRI scanner (Philips 3.0 T Achieva: Philips Healthcare, Andover, MA, United States) was used to acquire 3D T1 turbo field-echo MRI scans. Parameters were as follows: sagittal slice thickness, 1.0 mm with 50% overlap; and matrix size of 240 × 240 pixels reconstructed to 480 × 480 over a field view of 240 mm. Three-dimensional segmentation masks were obtained from the CIVET anatomical pipeline (version 2.1.0) for automated structural image analysis (Zijdenbos et al., 2002). The cortical thickness in the CIVET was computed using the Euclidean distance between the linked vertices of the inner and outer cortical surfaces (Kim et al., 2005, 2021). The thickness of the cortical regions of interest (ROIs_Cth) were the gray matter of the frontal, temporal, parietal, and occipital lobes. We also measured the extracerebral CSF (eCSF) volumes, focusing on the eCSF in the vicinity of the gray matter in the frontal, temporal, parietal, and occipital regions; the anterior and posterior LV volumes; and the volumes near the hippocampal regions of the LVs (ROIs_CSFvol).

**Figure 1:**
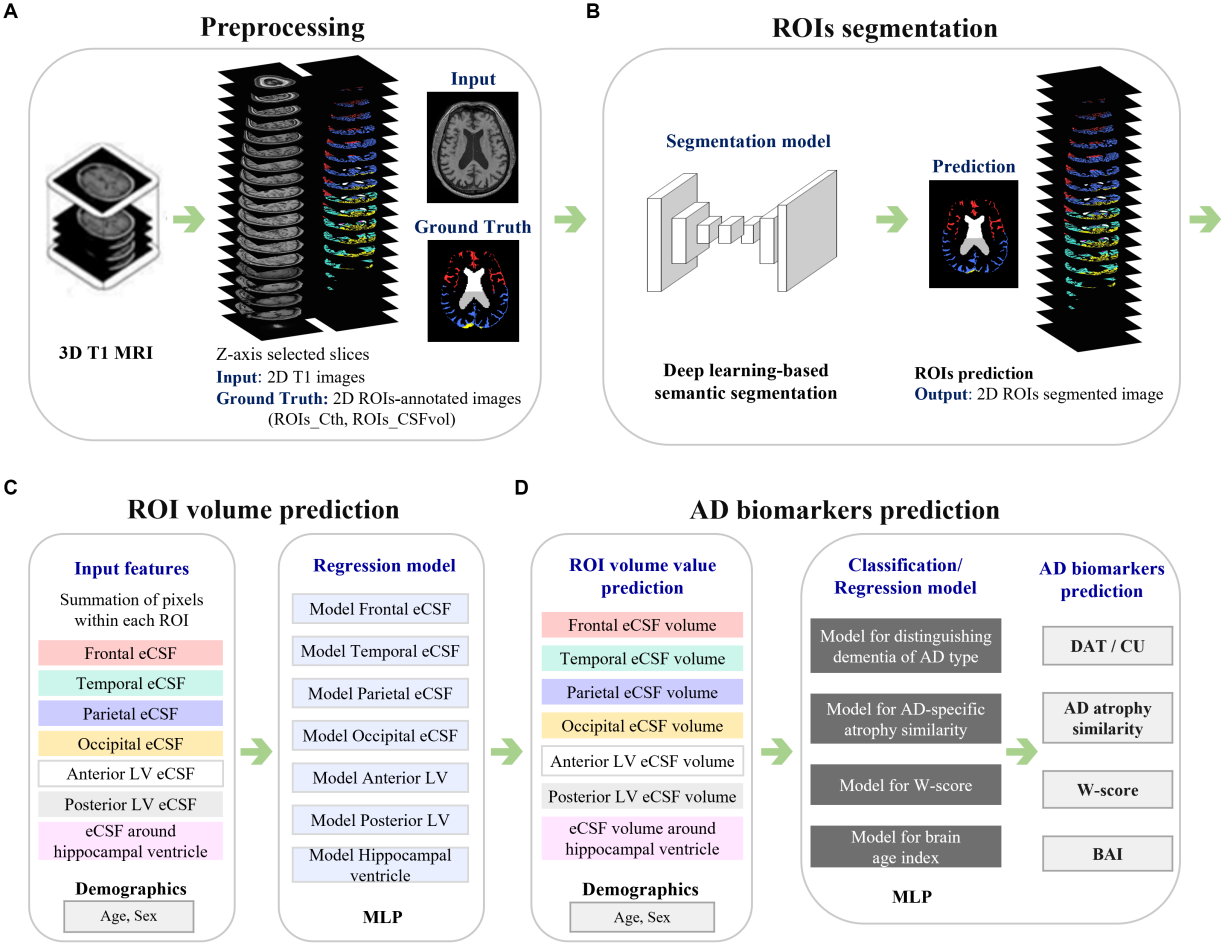
Framework of the study. The figure illustrates the analysis process of a system that automatically measures cortical thickness and CSF space from 2D MR images and predicts biomarkers related to Alzheimer’s disease. Panel **(A)** presents the preprocessing step, with 3D ROI annotations derived from 3D T1 MRI, leading to the acquisition of corresponding 2D images. Panel **(B)** shows the process of automatically segmenting ROIs in 2D MR images using deep learning techniques. Panel **(C)** represents the process of predicting the volume values for each ROI based on the segmented results from the images. Panel **(D)** demonstrates the use of the calculated volume values in predicting biomarkers related to Alzheimer’s disease. MRI, magnetic resonance imaging; ROIs, regions of interest; ROIs_Cth, ROIs of cortical thickness; ROIs_CSFvol, ROIs of cerebrospinal fluid space volume; eCSF, extracerebral cerebrospinal fluid space; LV, lateral ventricle; MLP, multilayer perceptron; AD, Alzheimer’s disease; DAT, dementia of Alzheimer’s type; CU, cognitively unimpaired; BAI, brain age index.

Figure 1 illustrates the framework used in this study. For preprocessing (Figure 1A), 20 of the 480 axial slices were selected from the 3D T1 images to match the image view acquired from the 2D MRI scan. Specifically, we extracted axial view 2D T1 images from 3D T1 images by selecting one image every 15 slices, as there were not many participants who had both 3D and 2D T1 images acquired simultaneously. Sampling was conducted representatively for some subjects, and the slice numbers that appeared similar to the 2D T1 images view were identified. We ensured that the entire head was included by confirming the top and bottom slices of the head. Then, Z-score normalization was applied to minimize brightness and contrast variations among the input 2D images. Two-dimensional label images in the axial view were also extracted from the CIVET 3D label mask images, where the label slice indices were identical to the selected MRI slice indices. After verifying the 2D label mask images, an image preprocessing technique of closing, with a kernel size of 5, was applied to smooth the noisy components in the masks. Data preprocessing steps were reviewed together with physicians, and all processed image files were stored and utilized in *Nifti* format.

### Deep learning-based segmentation for the 2D T1 images

Convolutional Neural Network-based deep learning models were developed to segment the ROIs (Figure 1B). For the image deep-learning semantic segmentation task of 2D T1 images, 980 cases with dimensions of 360, 480, and 480, corresponding to the x, y, and z axes, were selected from the 3D image format. Ultimately, a size of 480 by 360 for axial 2D images was used for the developed model. Image augmentation was applied for deep learning performance: axial MR images were randomly flipped in the horizontal direction, and brightness was adjusted in the range of-50 to 50. The physicians agreed to apply these preprocessing steps and use those as training data.

In the segmentation model, Inception-v3 based convolutional layers were employed for feature extraction (Szegedy et al., 2016), followed by the addition of deconvolutional layers. Skip connections were also implemented, linking each convolutional layer with its corresponding three deconvolutional layer to enhance detailed capture (Park et al., 2021). During training, 5-fold cross-validation was applied and the model was optimized using the Adam optimizer. The loss function employed was sparse softmax cross-entropy, and ReLU was utilized as the activation function. Additionally, L2 regularization was applied to prevent overfitting. The development of deep-learning network models was carried out using a Python 3.8 environment (Python Software Foundation), and the TensorFlow library was utilized for training the models.

The segmentation performance was evaluated by measuring the Dice Similarity Coefficient (DSC) between the ground truth and the prediction areas (model-based, automatically determined region). The DSC can be expressed in terms of True Positives (TP), False Positives (FP), and False Negatives (FN) as follows: DSC = 2 × TP / (2 × TP + FP + FN). The model was trained using a graphics processing unit (NVIDIA RTX A6000). The parameters were determined via grid search with a batch size of 2 and 4, dropout rates of 0.4, 0.5, and 0.6, learning rates of 1e-3, 1e-4, and 1e-5, and weight decays of 1e-4 and 1e-3. Batch normalization (Ioffe and Szegedy, 2015) and mean subtraction were used to prevent internal covariate shifts.

### Quantification of cortical thickness and CSF volume from 2D T1 images

The sum of the annotated areas from CIVET was used as a feature for deep learning models to train the relation between the annotated areas and the corresponding cortical thickness or volume of CSF spaces (Figure 1C). The total number of segmented pixels for each ROI was summed from a stack of segmentation results for each participant. Independent regression models were trained for each ROI using each participant’s features, including the ROI summation result, age, and sex information. The model was based on a Multi-Layer Perceptron (MLP) algorithm, where the ground truth for the model was the cortical thickness (Cth_3D) or CSF volume (CSFvol_3D) of the ROIs acquired from the 3D T1 images using the CIVET pipeline.

For the development of MLP models, experiments were conducted to determine optimal hyperparameters using a grid search with batch sizes of 16, 48, 64, 68, and 96; dropout rates of 0.3, 0.4, and 0.5; learning rates of 3e-4 and 3e-3; weight decays of 1e-4 and 1e-3; first hidden layers of 16, 32, 64, and 128; and second hidden layers of 4, 8, 16, 32, and 64. The models were developed using the PyTorch framework (Paszke et al., 2019). Ten times repeated 10-fold cross-validation was performed with the development dataset (*n* = 924). The best model, selected based on the minimum root mean square error within the optimal hyperparameter sets, was then evaluated on the test dataset (n = 196). We applied the best model for each ROI to predict the cortical thickness or CSF volume from the deep-learning-based segmentation results.

### Classifiers distinguishing DAT from CU and prediction of AD-specific atrophy similarity

Figure 1D provides a schematic overview of the development of the AD biomarker prediction model. Each model for the AD biomarkers was trained using a development dataset, applying 10-times repeated 10-fold cross-validation. After training, the best-performing model was selected and tested using an independent test set (Park et al., 2022).

Initially, classification models were developed to distinguish DAT from CU using MLP. The hyperparameter grid search was configured in the same manner as in the previous regression experiments. In the model training session, the input features included the CSF volume of the ROIs as well as age and sex. The performance was measured in terms of the area under the receiver operating characteristic curve (AUC) and the area under the precision-recall curve (AUPRC).

The AD-specific atrophy similarity measure quantitatively indicates the degree to which the brain observed in an individual’s brain image resembles an AD. Methods based on machine learning have been proposed for calculating AD-specific atrophy similarity (Lee et al., 2018a). In this study, the ‘AD-specific atrophy similarity’ is measured using a continuous value between 0 and 1 obtained from the DAT classification model. During training, DAT was mapped to 1 and CU to 0. An optimal threshold was then applied to distinguish between DAT and CU in the final stage. The continuous values generated, which approximated 1 for DAT cases, were used as AD-specific atrophy similarity.

### Prediction of W-scores and BAI using CSF volumes

Using the CSF volumes in the seven ROIs relative to the healthy control group, W-scores were computed for each participant. This metric is akin to z-scores but is modified for particular covariates. A previous investigation employed W-scores to encapsulate discrepancies in pathological characteristics between patient cohorts and control groups in neuroimaging (La Joie et al., 2012). In this study, we used age and sex as covariates in a multiple linear regression model to calculate the expected volume of the CSF space in each ROI. We recruited an isolated cohort of 109 CU individuals from the SMC. The W-score is calculated as follows:


$$W‐\mathrm{score}=\frac{V_{CSF}-E_{CU}\left(A,S\right)}{\sigma_{CU}}$$

where $V_{CSF}$ is the participant’s CSF space volume, $E_{CU}\left(A,S\right)$ is he expected CSF space volume in the CU group for the participant’s age (A) and sex (S), and $\sigma_{CU}$ is the standard deviation of the residuals in the CU group. A positive W-score denotes a volumetric increase in the CSF in certain brain regions. In the present study, W-scores were computed from the CSF volumes at ROIs_ CSFvol based on the 3D T1 (CSFvol_3D) and 2D T1 images (CSFvol_2D). The correlation coefficients *r* were calculated between the W-scores of CSFvol_3D and CSFvol_2D.

In addition, individual BAIs were calculated from the seven ROIs_CSFvol. The ground truth for brain age was estimated using Statistical Parametric Mapping 12 software, and a regression model based on MLP was developed to predict BAIs. The input features for the MLP model were the CSFvol_3D as well as age and sex, and the hyperparameter grid search was set up similarly to the previous experiments. A 10-fold cross-validation was repeated 10 times using the development dataset (n = 896) and evaluated with the test datasets (*n* = 187). Correlation coefficients *r* were calculated between BAI values from CSFvol_3D and CSFvol_2D.

### Statistical analyses

We used the Student’s *t*-test for normally distributed continuous variables and the Mann–Whitney U test for non-normally distributed variables to compare the two groups. The chi-square test was used to examine the associations between categorical variables. We considered *p* < 0.05 to be statistically significant. To evaluate the statistical differences between the AUCs in the classification task, we conducted the DeLong’s test (DeLong et al., 1988). We performed Pearson correlation analyses and Bland–Altman analyses to investigate the relations between 3D T1 and 2D T1 measurements of cortical thickness and CSF volume, AD-specific atrophy similarity, W-scores, and BAI. Statistical analyses were performed using the *scipy* package of Python 3.8.

## Results

### Clinical characteristics

Table 1 presents the demographic and clinical characteristics of the participants. Among the 924 participants in the development dataset, 478 (51.7%) were diagnosed with CU, and 446 (48.3%) were diagnosed with DAT. The mean age was 68.3 ± 11.6 (mean ± SD) years for the CU group and 70.3 ± 9.8 years for the DAT group. The proportions of females were 59.0 and 58.7% in the CU and DAT groups, respectively. The proportion of *APOE* ε4 carriers was 25.3% among the CU participants and 52.9% among those with DAT. No statistically significant differences were observed between the model development and test dataset.

**Table 1:** Demographics of participants.

	Development dataset				Test dataset				*p*-value^†^
	Total	CU	DAT	*p*-value	Total	CU	DAT	*p*-value	
	*N* = 924	*N* = 478	*N* = 446		*N* = 196	*N* = 113	*N* = 83		
Age, years	69.2 ± 10.8	68.3 ± 11.6	70.3 ± 9.8	0.087	67.9 ± 11.3	67.9 ± 11.1	67.9 ± 11.7	0.640	0.086
Female, *N* (%)	542 (58.7)	282 (59.0)	262 (58.7)	1.000	115 (58.7)	71 (62.3)	44 (52.4)	0.218	1.000
Education, years	11.6 ± 4.7	12.0 ± 4.6	11.2 ± 4.8	0.004	12.1 ± 5.1	12.1 ± 4.9	12.2 ± 5.4	0.634	0.098
*APOE ε*4 carriers, *N* (%)	357 (39.7)	121 (25.3)	236 (52.9)	<0.001	60 (31.4)	28 (24.6)	32 (38.1)	0.014	1.000
MMSE	23.8 ± 6.1	28.1 ± 1.9	19.1 ± 5.6	<0.001	24.2 ± 5.9	28.1 ± 2.1	18.9 ± 5.3	<0.001	0.338

The values are expressed as mean ± standard deviation or number (percentage).CU, cognitively unimpaired; DAT, dementia of Alzheimer’s type; SD, standard deviation; APOE ε4, apolipoprotein E ε4; MMSE, Mini-Mental State Exam. †Comparisons between development and test dataset.

### Performances of segmentation

The segmentation results of each ROIs_Cth measured in the 5-fold averaged DSC were as follows: 0.816 (95% Confidence Interval [CI]: 0.812–0.820) for frontal Cth, 0.793 (0.790–0.797) for temporal Cth, 0.777 (0.773–0.783) for parietal Cth, and 0.720 (0.712–0.728) for occipital Cth. The average DSC values of the CSF space segmentation were 0.874 (0.870–0.878) for the anterior LV, 0.852 (0.847–0.857) for the posterior LV, and 0.637 (0.628–0.646) for the region around the hippocampal ventricle. The average DSC values for the frontal, temporal, parietal, and occipital eCSF were 0.640 (0.625–0.655), 0.524 (0.508–0.540), 0.632 (0.618–0.646), and 0.502 (0.485–0.519), respectively (95% CI for all values). The optimized hyperparameters found through experimentation are as follows: batch size of 4, dropout rate of 0.5, learning rate of 1e-4, and, weight decay of 1e-5. Supplementary Figure S2 shows the 2D T1 images (left), corresponding ground-truth images (middle), and predicted images (right).

### Correlations between Cth_3D and Cth_2D, and between CSFvol_3D and CSFvol_2D

**Figure 2:**
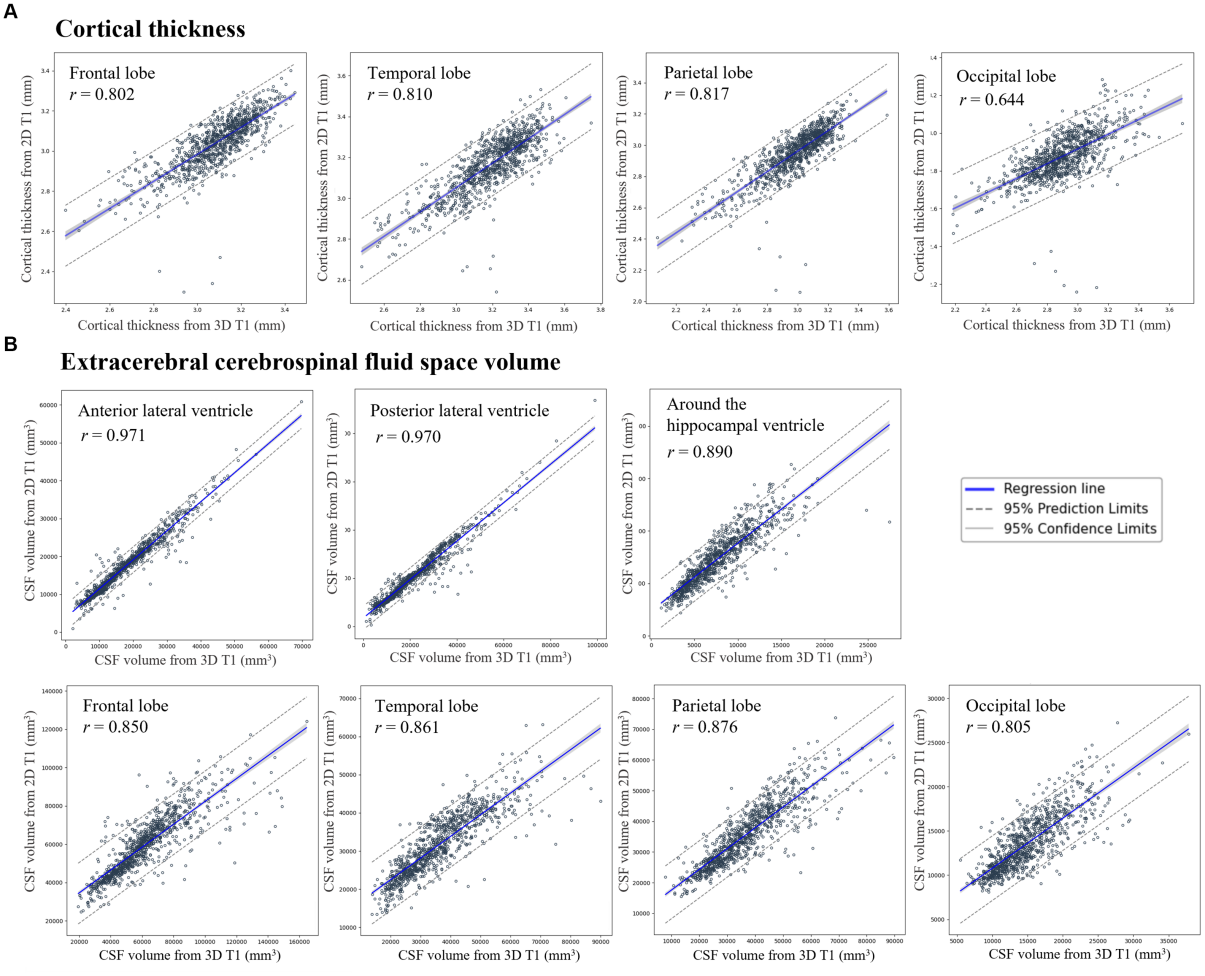
Correlation of **(A)** cortical thickness and **(B)** extracerebral cerebrospinal fluid space volume between 3D T1 and 2D T1 across regions of interest. Scatter plots show correlations for **(A)** cortical thickness (mm) in the frontal, temporal, parietal, and occipital lobes, and **(B)** extracerebral cerebrospinal fluid (eCSF) space volume (mm3) in the vicinity of the gray matter in the frontal, temporal, parietal, and occipital regions, the anterior and posterior lateral ventricle volumes, and volumes nearby hippocampus regions. Regression lines and 95% confidence intervals compare measurements from 3D T1 (x-axis) to 2D T1 (y-axis). 3D, three-dimensional; 2D, two-dimensional; MR, magnetic resonance imaging; CSF, cerebrospinal fluid.

Cth_2D was highly correlated with Cth_3D (Figure 2A) with correlation coefficient *r* of 0.802 (0.778–0.823) for frontal gray matter, 0.810 (0.787–0.830) for temporal gray matter, 0.817 (0.795–0.837) for parietal gray matter, and 0.644 (0.606–0.680) for occipital gray matter. Moreover, CSFvol_2D was highly correlated with CSFvol_3D (Figure 2B), with correlation coefficients *r* of 0.850 (0.832–0.866) for frontal eCSF, 0.861 (0.844–0.876) for temporal eCSF, 0.876 (0.860–0.890) for parietal eCSF, 0.805 (0.782–0.826) for occipital eCSF, 0.971 (0.967–0.974) for anterior LV, 0.970 (0.966–0.973) for posterior LV, and 0.890 (0.877–0.903) for the region surrounding the hippocampal ventricle. The optimized hyperparameters of the lateral ventricle were as follows: first hidden layer of 128 nodes, second hidden layer of 16 nodes, a batch size of 64, a dropout rate of 0.3, a learning rate of 3e-3, and a weight decay of 1e-4. Because the correlation coefficient *r* values between CSFvol_3D and CSFvol_2D were higher than those between Cth_3D and Cth_2D, subsequent analyses (including distinguishing DAT from CU, AD-specific atrophy similarity, W-scores, and BAI) were conducted using CSFvol_2D but not Cth_2D.

In order to compare the ‘true 2D T1 images’ and the ‘2D T1 images derived from 3D T1 images,’ we obtained an independent dataset of 364 participants (170 CU and 194 DAT) with both true 2D T1 and 3D T1 images (Supplementary Table S1). The CSFvol_2D from the 3D T1 images was highly correlated with the true 2D T1 images (Supplementary Figure S3), with correlation coefficients *r* of 0.815 for frontal eCSF, 0.938 for temporal eCSF, 0.878 for parietal eCSF, 0.798 for occipital eCSF, 0.998 for anterior LV, 0.997 for posterior LV, and 0.988 for the region surrounding the hippocampal ventricle.

### Performances of DAT classifiers and AD-specific atrophy similarity based on CSFvol_2D

The performance of the classifier based on CSFvol_3D exhibited an AUC of 0.905 and an AUPRC of 0.891. Similarly, the classifier’s performance based on CSFvol_2D demonstrated high accuracy, comparable to that of the CSFvol_3D-based classifier, with the model inputs yielding an AUC of 0.873, an AUPRC of 0.849, a sensitivity of 0.819, and a specificity of 0.761. The DeLong et al. (1988) test was performed to compare the AUCs of CSFvol_3D and CSFvol_2D. The obtained *p*-value was 0.053, indicating no significant difference in the analysis results between the conventional 3D T1-based analysis and the proposed 2D T1-based deep learning analysis. The optimal hyperparameters for the classifier were as follows: 128 nodes in the first hidden layer, 16 nodes in the second hidden layer, batch size of 64, dropout rate of 0.5, learning rate of 3e-4, and weight decay of 1e-4.

We conducted an error analysis of the classification results, and the findings are as follows: For false positives, where the clinical diagnosis is CU but the model predicted DAT, the CSF volume values were generally predicted to be lower compared to the true positive cases due to the poor image segmentation, and the average age was higher (75.0 ± 5.4 vs. 64.5 ± 11.1). For false negatives, where the clinical diagnosis is DAT but the model predicted CU, the CSF volume values were generally predicted to be higher compared to the true negative cases, and the average age was also higher (73.7 ± 10.5 vs. 67.5 ± 11.3).

**Figure 3:**
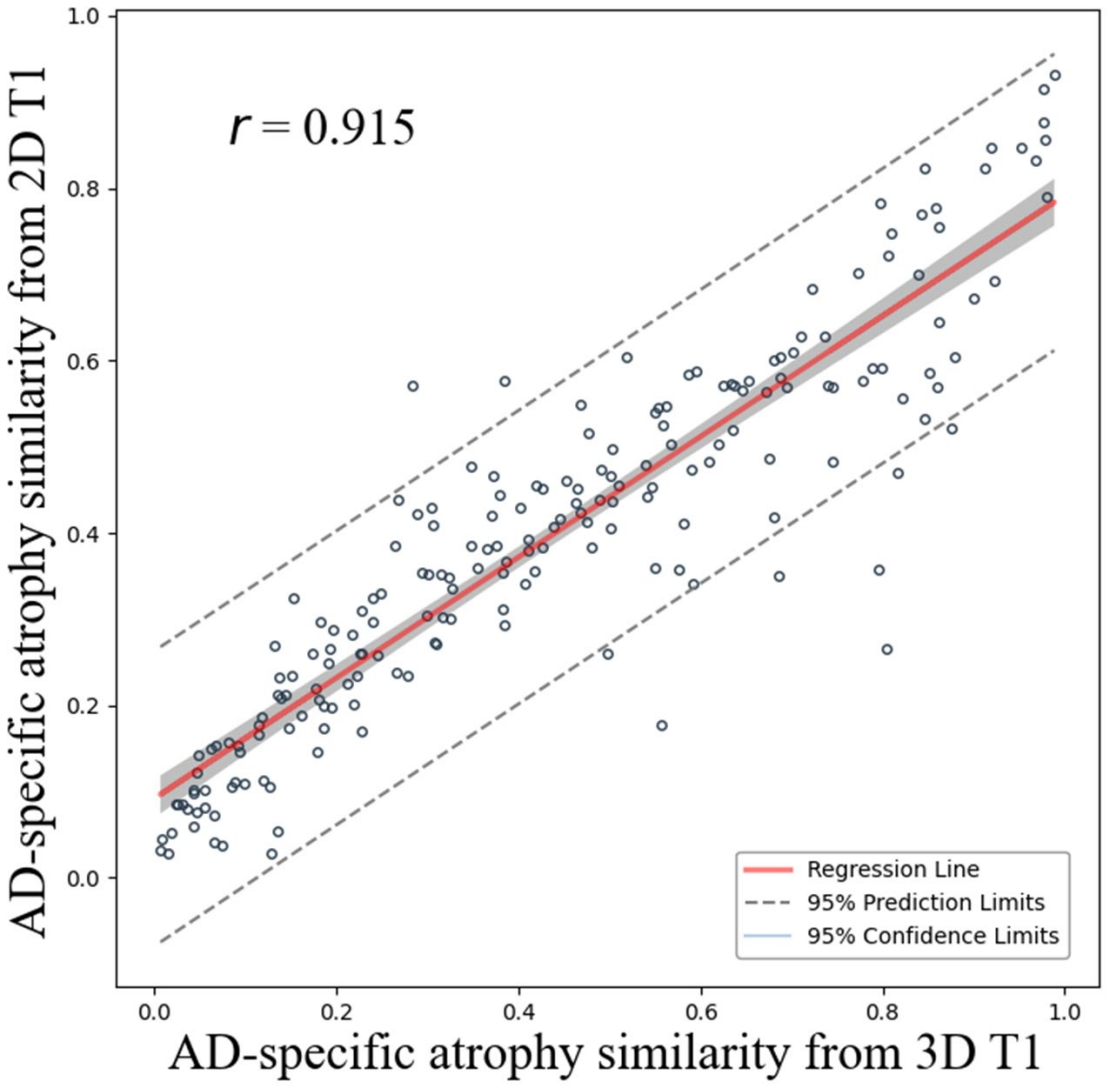
Correlation of AD-specific atrophy similarity between 3D T1 and 2D T1. Scatter plots compare AD-specific atrophy similarity measures derived from cerebrospinal fluid space volume in 3D T1 (x-axis) and 2D T1 (y-axis) with regression lines and 95% confidence intervals.

The correlation coefficient *r* between AD-specific atrophy similarity based on CSFvol_3D and that based on CSFvol_2D was 0.915 (0.889–0.935) (Figure 3), indicating a high degree of correlation. The Bland–Altman plot is presented in Supplementary Figure S4A.

### W-scores and BAI based on CSFvol_2D

**Figure 4:**
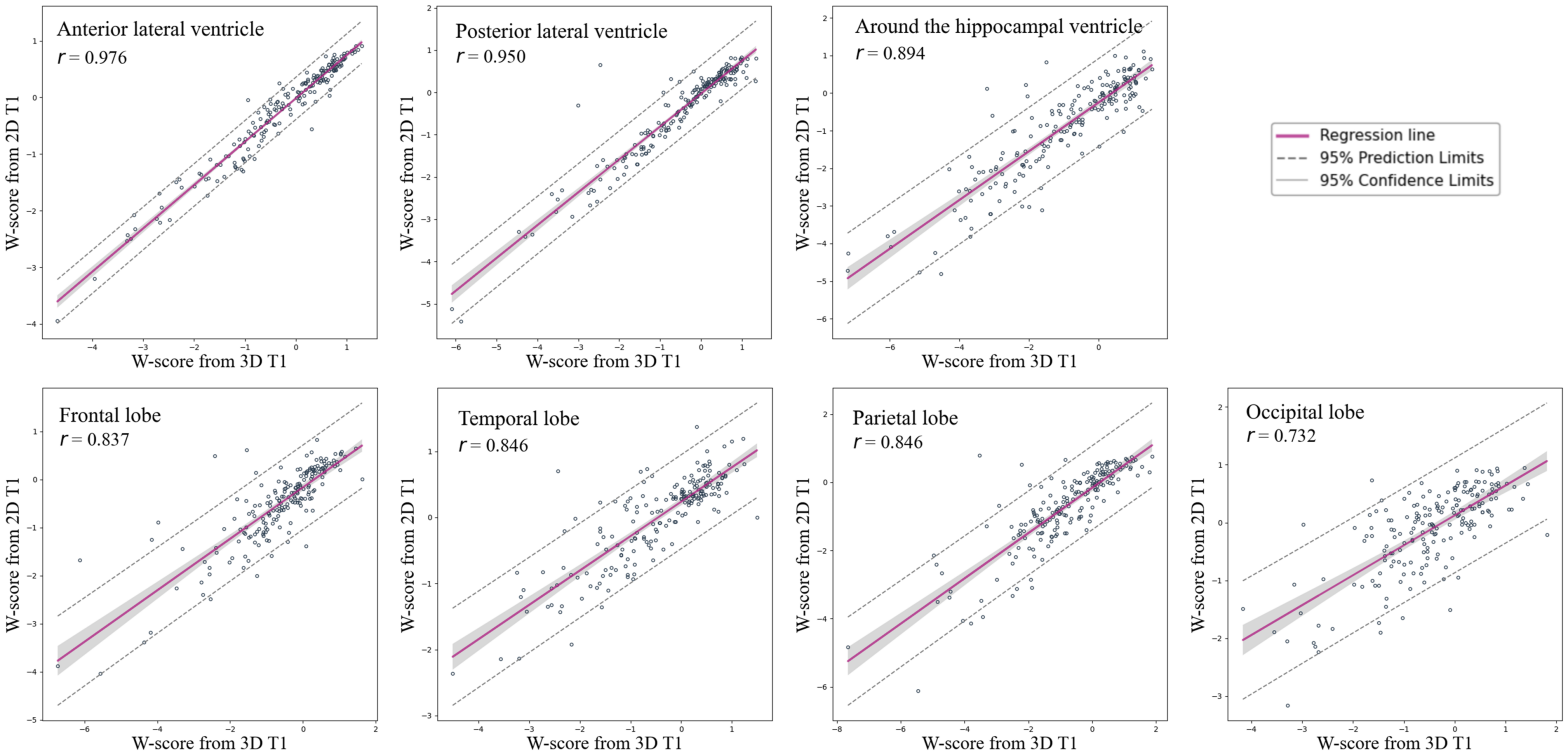
Correlation of W-scores for brain regions between 3D T1 and 2D T1 images. Scatter plots and regression lines with 95% confidence intervals illustrate the correlation of W-scores, indicating brain atrophy, between 3D T1 and 2D T1 images across various brain regions, including the frontal lobe, temporal lobe, parietal lobe, occipital lobe, anterior lateral ventricle, posterior lateral ventricle, and the region around the hippocampal ventricle. 3D, three-dimensional; 2D, two-dimensional.

Figure 4 shows the correlation between W-scores calculated using CSFvol_3D and CSFvol_2D. The correlation coefficients *r* for the W-scores in the LV were the strongest at 0.976 (0.969–0.982) for the anterior LV, and 0.950 (0.935–0.962) for the posterior LV. The volume around the hippocampal ventricle also showed a strong correlation, with a correlation coefficient *r* of 0.894 (0.862–0.919). The eCSF volumes in the frontal, temporal, parietal, and occipital regions also exhibited high correlation coefficients *r* of 0.837 (0.790–0.875), 0.846 (0.801–0.882), 0.846 (0.801–0.882), and 0.732 (0.659–0.791), respectively.

**Figure 5:**
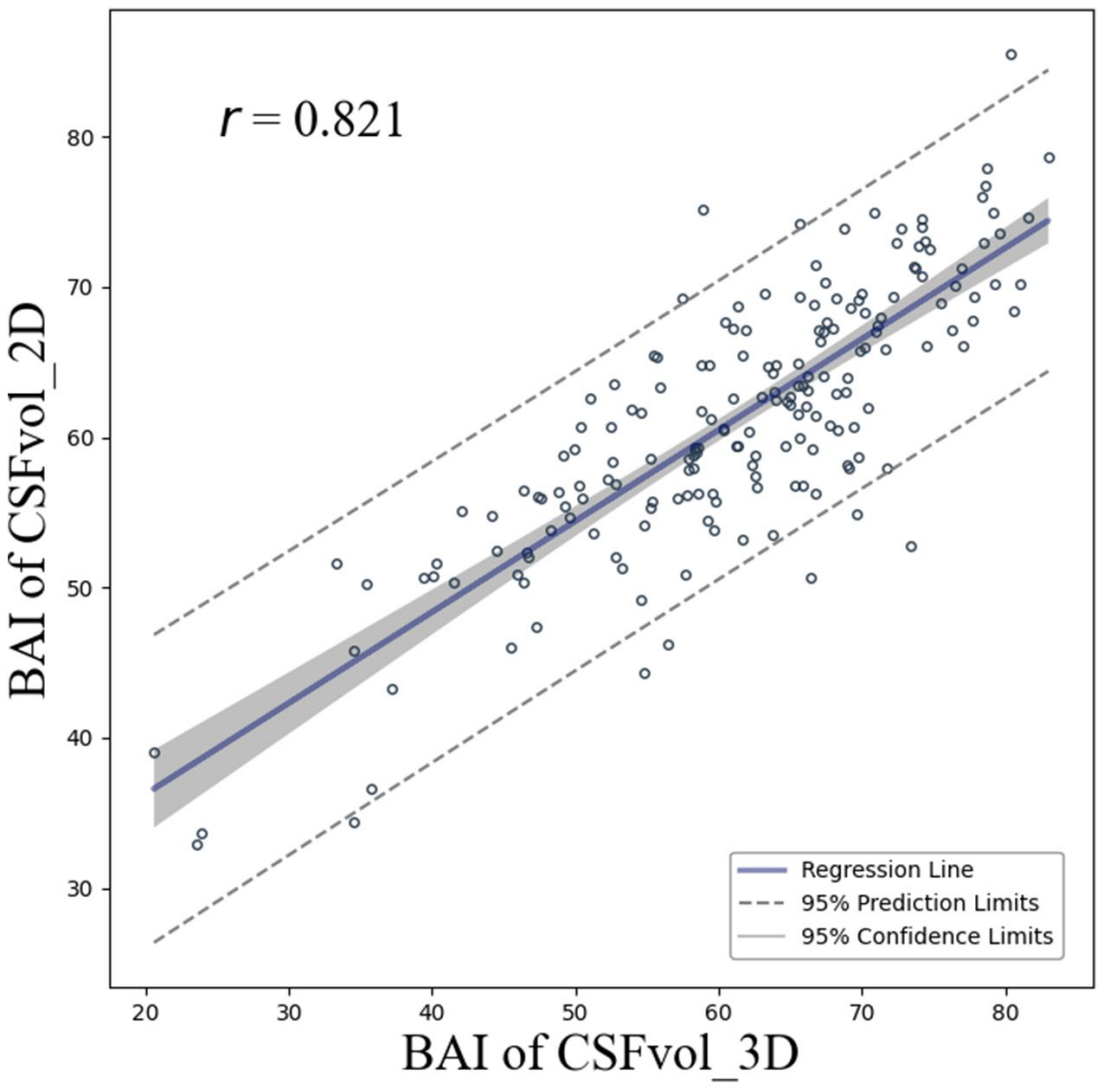
Correlation of brain age index between 3D T1 and 2D T1 images. Scatter plots compare brain age index derived from cerebrospinal fluid space volume in 3D T1 (x-axis) and 2D T1 (y-axis) with regression lines and 95% confidence intervals. BAI, brain age index; 3D, three-dimensional; 2D, two-dimensional.

We assessed the correlation between the BAI calculated based on CSFvol_3D and BAI calculated based on CSFvol_2D (Figure 5). The correlation coefficient *r* between the two BAIs was 0.821 (0.768–0.863), and the Bland–Altman plot is presented in Supplementary Figure S4B. The optimal hyperparameters for the BAI model were: 128 nodes in the first hidden layer, 64 nodes in the second, batch size of 68, 0.5 dropout rate, learning rate of 3e-4, and weight decay of 1e-4.

### Summary of 2D T1 analysis results in comparison with 3D T1

Segmentation results showed that larger and simpler ROI masks achieved higher performance, with the best results in LV regions. In predicting quantitative measures like cortical thickness or volume from segmented regions, CSF space models (LV, eCSF) outperformed cortical thickness models. For the model distinguishing between DAT and CU, the 2D-based analysis demonstrated high performance (AUC 0.873), showing comparable accuracy to the 3D-based standard method (AUC 0.905) for measuring brain atrophy.

In DAT-related biomarkers such as AD-specific atrophy similarity, W-score, and BAI, the 2D T1 analysis results were highly correlated with 3D T1 results. Notably, higher eCSF volume prediction performance corresponded with higher W-score prediction performance for each ROI.

## Discussion

In this study, we developed deep learning-based models that utilize CSF volumes from 2D T1-weighted images. We validated the clinical utility of our algorithms by differentiating DAT from CU participants, predicting AD-specific atrophy similarities, estimating W-scores for brain atrophy, and calculating BAIs relative to age and sex. Our major findings are as follows. First, the CSF volumes based on 2D T1 images were highly correlated with those based on 3D T1 images. Second, our newly developed algorithms using 2D T1 image-derived CSF volumes showed excellent performance in differentiating DAT from CU and very high correlations in AD-specific atrophy similarity, W-scores for brain atrophy, and BAIs compared with those based on 3D T1 images. Taken together, our findings suggest that deep learning-based models based on CSF volumes from 2D T1 images may be a viable alternative to 3D T1 images for assessing brain atrophy in clinical settings. The clinical utility of our newly developed algorithms was validated in various settings with high accuracy, comparable to that achieved with 3D T1 image-based algorithms. Using accessible and cost-effective 2D T1 images for quantifying brain atrophy and AD classification enables earlier detection of neurodegenerative changes, leading to timely intervention and better management of atrophy and cognitive decline.

Our first major finding was that the CSF volumes based on 2D T1 images (CSFvol_2D) were significantly correlated with those based on 3D T1 images (CSFvol_3D). In clinical settings, the assessment of brain atrophy involves the evaluation of enlarged CSF volumes, indicative of the loss of adjacent gray matter and white matter. Traditionally, clinicians have relied on visual assessment scales from 2D T1 MR images or CT scans by utilizing the enlarged CSF regions, including the LVs, sulcal widening between the gyri, and the width of the temporal horn adjacent to the hippocampus (Koedam et al., 2011; Harper et al., 2015). However, these visual assessment scales do not show high concordance rates among clinicians, and there are no quantitative methods for 2D T1 images. Therefore, our findings underscore the reliability of CSFvol_2D as an effective surrogate for complex and time-intensive 3D T1 measurements. Furthermore, CSFvol_2D could provide a more accessible and economically viable alternative without compromising diagnostic accuracy for assessing brain atrophy.

In the present study, we applied Fully Convolutional Network-based deep learning techniques to 2D MR for automatic brain segmentation, resulting in high segmentation performance (particularly in the anterior and posterior LV, with DSCs of 0.874 and 0.852, respectively). Previous methods for quantifying brain atrophy often use 3D T1 images with CIVET or FreeSurfer software to measure cortical thickness. Recently, deep learning-based approaches have emerged (Rebsamen et al., 2020), showing a Pearson correlation of *r* = 0.740 with FreeSurfer across frontal, temporal, parietal, and occipital lobes using 3D T1 (Cth_3D). Our study achieved a higher correlation of *r* = 0.768 (Cth_2D averaged) with CIVET using 2D T1 images and a deep learning model. Furthermore, our study showed that the correlations between results based on 2D T1 images and those based on 3D T1 images were higher for CSF volume than for cortical thickness. Our findings might be explained by the fact that the differences in intensities between gray matter and CSF or between white matter and CSF (the main distinct features in our models of CSF volumes) were more pronounced than the differences in intensities between gray matter and white matter (the main distinct features in our models of cortical thickness). That is, the more distinct differences between features were more reflective of the results based on 3D T1 images into the results based on 2D T1 images in CSF volumes than in cortical thickness, which in turn resulted in higher correlation in CSF volumes.

Notably, the LV exhibited the highest Pearson correlation coefficients among the CSF volumes (anterior LV, 0.971; posterior LV, 0.970). The large area of the LV relative to other brain ROIs and its comparatively simple shape facilitate distinction from other brain structures. This high degree of correlation is noteworthy because ventricular dilatation (particularly of the frontal, occipital, and temporal horns of the LV) is a critical metric for assessing cerebral atrophy (Pasquier et al., 1996). Additionally, the volume around the hippocampal ventricle showed a strong correlation between CSFvol_2D and CSFvol_3D. The temporal horn of the LV is crucial for evaluating medial temporal lobe atrophy in probable AD (Scheltens et al., 1992; Kim et al., 2014). Sulcal widening between the gyri in each lobe was used as an indicator of lobar atrophy. Different types of dementia display unique patterns of brain atrophy. AD is typically characterized by temporoparietal atrophy, whereas frontotemporal dementia is characterized by frontotemporal atrophy. Thus, ROIs_CSFvol, including the anterior LV, posterior LV, volume around the hippocampal ventricle, and eCSFs in each lobe, might be one of the most important features for differentiating the causes of dementia. Further studies are required to determine whether our newly developed models are effective in distinguishing between the causes of various types of dementia.

Our second major finding was that our MLP model demonstrated good performance in differentiating DAT from CU participants, achieving AUC values of 0.873 for classifiers based on CSFvol_2D and 0.905 for conventional classifiers based on CSFvol_3D. Our previous classifiers, based on Cth_3D, showed an accuracy of 91.1% in differentiating DAT from CU (Lee et al., 2018a). In addition, AD-specific atrophy similarity measures derived from CSFvol_2D highly correlated with those obtained from CSFvol_3D. Our AD-specific atrophy similarity measure represents the similarity of the cortical atrophy pattern of an individual patient to that of a representative patient with AD, determined using a well-defined AD cohort. In our previous study (Lee et al., 2018a), the AD-specific atrophy similarity measure showed promising results at the individual level, not only facilitating the early prediction of AD but also distinguishing between brain and clinical trajectories in patients with DAT. Therefore, our findings underscore the potential of quantitative analyses based on CSFvol_2D, especially the LV, volumes around the hippocampal ventricle, and eCSF for the precise diagnosis of DAT and early initiation of therapeutic interventions.

Our final major finding was that the brain atrophic W-scores, after adjusting for age and sex, derived from CSFvol_2D were highly correlated with those from CSFvol_3D. As aging progresses, brain atrophy occurs at a mean volume reduction rate of 0.5% per year after the age of 40 (Fotenos et al., 2005; Lee et al., 2018b). In addition, changes in brain atrophy have been shown to occur differently depending on sex (Lee et al., 2018b; Kim et al., 2019; Suzuki et al., 2019). Thus, our age-and sex-adjusted brain atrophic W-scores may help clinicians distinguish pathological brain atrophy from physiological age-related brain atrophy. The trajectory of brain atrophy throughout aging can be captured and translated into an individual’s brain age using machine-learning algorithms. Brain age serves as an indicator of overall brain health as it allows for individual-level inferences rather than group-level assessments. Furthermore, an increased BAI is predictive of worse cognitive trajectories (Gaser et al., 2013; Wang et al., 2019). In the present study, the BAIs based on CSFvol_2D correlated strongly with those based on CSFvol_3D, suggesting that our newly developed BAI based on CSFvol_2D may assist clinicians in diagnosing and managing individuals with pathological brain atrophy.

The strength of our study lies in the innovative application of deep learning to the reconstruction of 2D T1 MR images for quantitative analysis. Several algorithms have been developed for classifying DAT, predicting AD-specific atrophy similarity, assessing brain atrophy W-scores, and estimating the BAI. However, the present study has some limitations. First, we used clinical criteria for DAT rather than AD biomarker-guided diagnosis. Further studies incorporating AD biomarker-guided diagnoses are required to develop algorithms to predict AD biomarkers. Second, the deployment of various deep learning architectures, particularly the most recent image segmentation models (Isensee et al., 2021; Ma et al., 2024), has not yet been explored. While this study utilized an MLP model for predicting AD biomarkers, it is possible to achieve higher accuracy by applying various machine learning techniques such as random forest and support vector machines, or by creating an ensemble model. Future research should consider evaluating the performance through the integration of models with iterative updates. Third, 20 axial slices selected from 3D T1 images, so there may be any information loss in this process. Additionally, the slice thickness of the ‘2D T1 images from 3D T1 images’ used in our study may differ from the typically acquired slice thickness in clinical practice, leading to lower generalizability of our results to common clinical settings. To match the difference in acquisition protocol, we extracted 20 slices with a 5 mm slice thickness from the 3D T1 images, as the ‘true 2D T1 images’ acquired at our center are obtained with 5 mm slice thickness and gaps between slices, resulting in approximately 20 slices. However, since 2D T1 images are used in clinical practice, the purpose of this study was to determine whether brain atrophy, which can only be measured with 3D T1 images, can be measured with 2D T1 images. This argument might be mitigated by our findings that brain atrophy measured with ‘2D T1 images from 3D T1 images’ is comparable to brain atrophy measured with ‘true 2D T1 images.’ Fourth, in our main analysis, we used ‘2D T1 images from 3D T1 images’ instead of the ‘true 2D T1 images.’ However, considering the high correlation between the ‘true 2D T1 images’ and the ‘2D T1 images from 3D T1 images,’ we expect the correlation for DAT/CU classification, AD-specific atrophy similarity, W-scores, and BAIs to be similarly high. Fifth, the ROIs we chose are relatively less granular than those used other methods, so they have not been fully validated to ensure they are regionally relevant to dementia. Thus, future research is needed to explore whether our methods are useful for distinguishing subtypes of dementia. Finally, the model was assessed using data from a single cohort. Incorporating larger datasets, potentially from multiple cohorts, is essential to ensure the robustness and generalizability of our findings. Thus, future studies should be conducted to see if the same results can be achieved using 2D T1 images from different vendors at different centers in different patient populations. Techniques related to image registration and domain adaptation may need to be applied during the implementation process. Nevertheless, our study provides valuable insights, demonstrating that deep learning-based quantitative analysis using 2D T1 images, a modality widely adopted in clinical practice, can be effective. Although there might be several challenges, including securing the necessary infrastructure such as the scanner settings and analysis platforms, providing adequate training for radiologists, and incorporating the approach into existing clinical workflows, addressing these challenges will be beneficial for clinical settings.

In conclusion, our study revealed that deep-learning analyses based on 2D T1 CSF volumes were highly correlated with those based on 3D T1 CSF volumes. Furthermore, our study demonstrates the feasibility of using deep-learning-based 2D T1 CSF volumes for the DAT classifier, AD-specific atrophy similarity, W-scores, and BAI, establishing 2D MR as a dependable, cost-effective, and accessible tool in clinical practice. Therefore, our findings contribute to the application of 2D MR quantitative analysis, especially for retrospective analysis of images acquired in 2D T1 and in settings with limited access to 3D imaging technology.

## Data availability statement

This study utilized BeauBrain Healthcare Morph’s image processing technology to examine brain atrophy and classify Alzheimer’s Disease using MR images. Data will be made available to qualified investigators upon reasonable request to the corresponding authors.

## Ethics statement

The studies involving humans were approved by Institutional Review Board of Samsung Medical Center. The studies were conducted in accordance with the local legislation and institutional requirements. The participants provided their written informed consent to participate in this study.

## Author contributions

CP: Conceptualization, Formal analysis, Funding acquisition, Methodology, Writing – original draft. YP: Data curation, Formal analysis, Writing – original draft. KK: Data curation, Formal analysis, Writing – original draft. SC: Data curation, Formal analysis, Writing – original draft. HK: Writing – review & editing, Investigation. DN: Writing – review & editing, Investigation. SS: Conceptualization, Funding acquisition, Investigation, Supervision, Writing – original draft. MC: Conceptualization, Funding acquisition, Supervision, Writing – original draft.
