# Supplementary material for: Deep learning-based quantification of brain atrophy using 2D T1-weighted MRI for Alzheimer’s disease classification
Source: Front Aging Neurosci. 2024 Aug 14;16:1423515. doi: 10.3389/fnagi.2024.1423515 (PMC11349618; doi:10.3389/fnagi.2024.1423515)
Supplement: Supplementary file 1 [file Data_Sheet_1.docx]

**
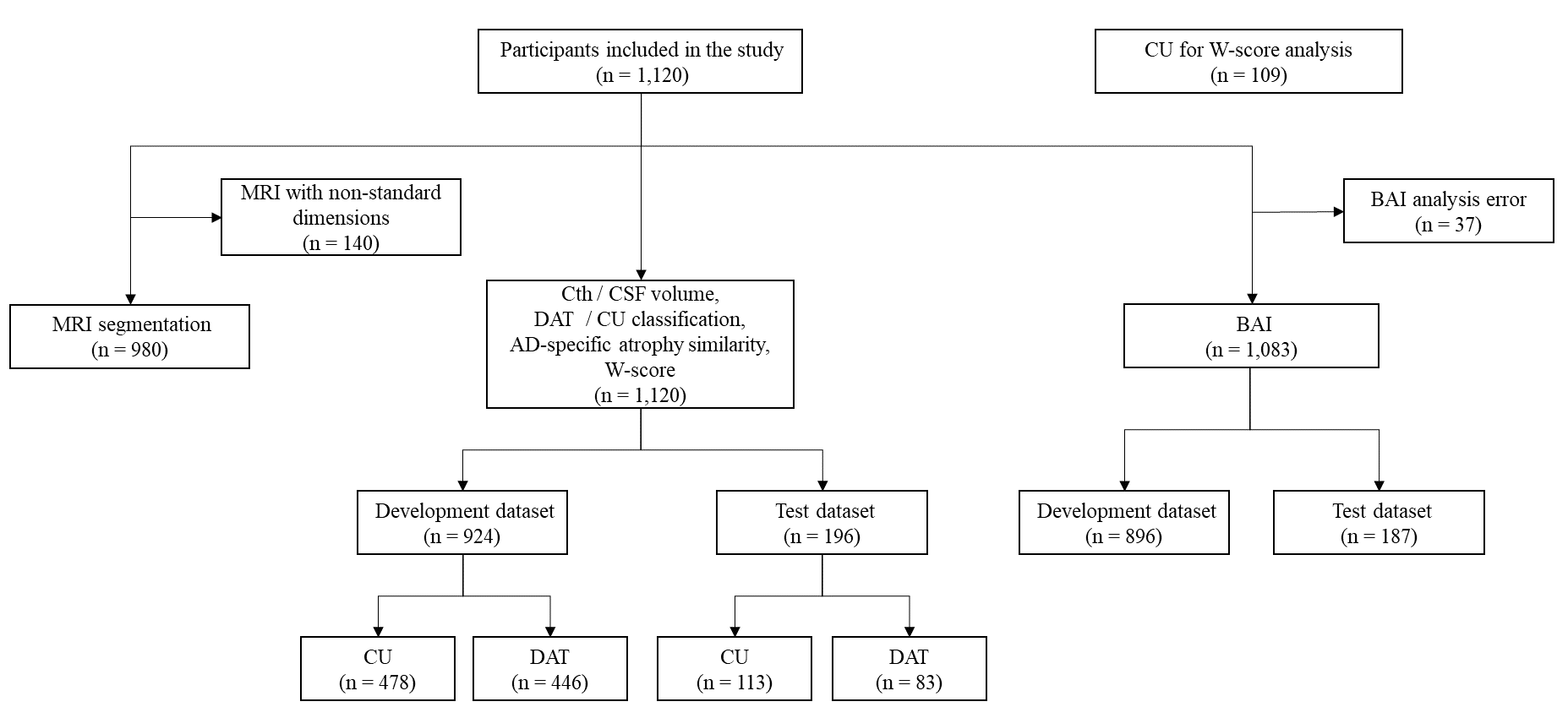
**

**Supplementary Figure 1. Flow diagrams of study population**

Abbreviations: CU, cognitively unimpaired; MRI, magnetic resonance imaging; Cth, cortical thickness; CSF, cerebrospinal fluid; DAT, dementia of Alzheimer's type; AD, Alzheimer’s disease; BAI, brain age index.


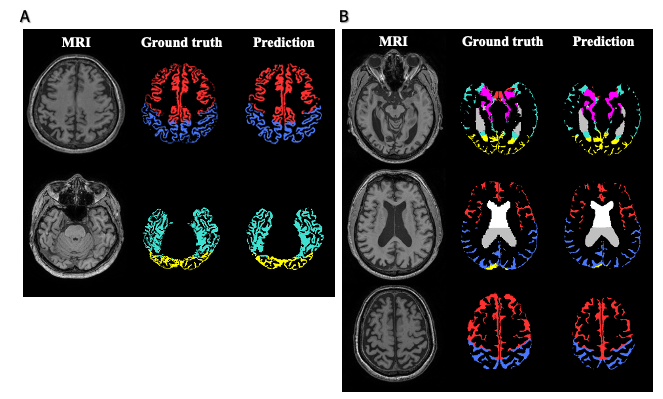


**Supplementary Figure 2**. **Segmentation of brain regions in 2D T1 MR.**

Left: 2D T1 MR images. Middle: the corresponding 3D label mask representing ground truth. Right: prediction results from deep-learning segmentation.

**(A)** Cortical thickness regions of interest (ROIs_Cth) are shown with frontal (red), temporal (green), parietal (blue), and occipital (yellow) lobes color-coded.

**(B)** Extracerebral cerebrospinal fluid (eCSF) space volumes (ROIs_CSFvol) are illustrated with frontal eCSF (red), temporal eCSF (green), parietal eCSF (blue), occipital eCSF (yellow), anterior lateral ventricle (white), posterior lateral ventricle (gray), and the region around the hippocampal ventricle (magenta).

Abbreviations: MRI, magnetic resonance imaging; 2D, two-dimensional; 3D, three-dimensional.

**
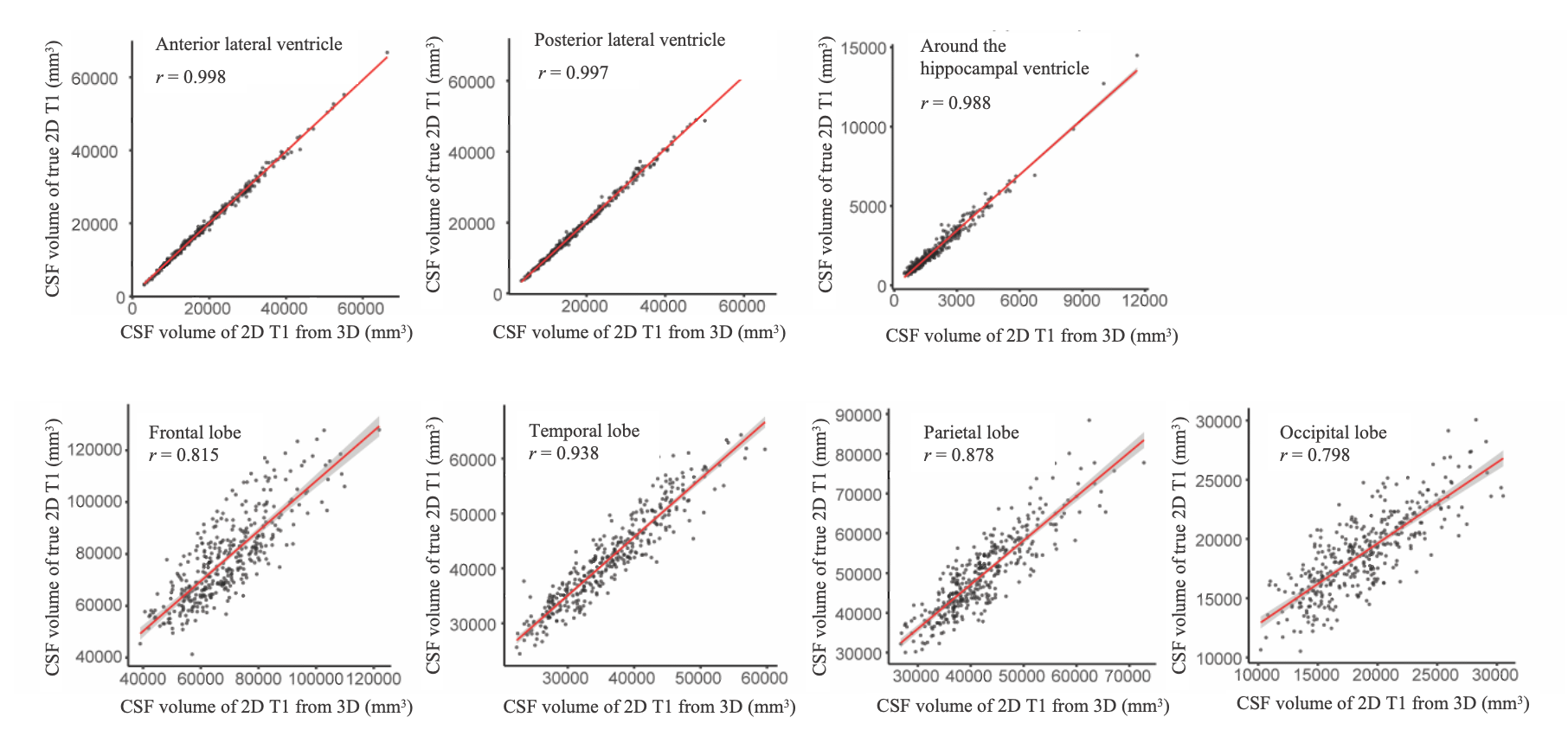
**

**Supplementary Figure 3. Correlation of extracerebral cerebrospinal fluid space volume between 2D T1 from 3D MR and true 2D T1 across regions of interest.**

Scatter plots show correlations for extracerebral cerebrospinal fluid space volume (mm^3^) in the vicinity of the gray matter in the frontal, temporal, parietal, and occipital regions, the anterior and posterior lateral ventricle volumes, and volumes nearby hippocampus regions.

Regression lines and 95% confidence intervals compare measurements of 2D T1 from 3D MR (x-axis) to true 2D T1 (y-axis).

Abbreviations: 3D, three-dimensional; 2D, two-dimensional; MR, magnetic resonance imaging; CSF, cerebrospinal fluid


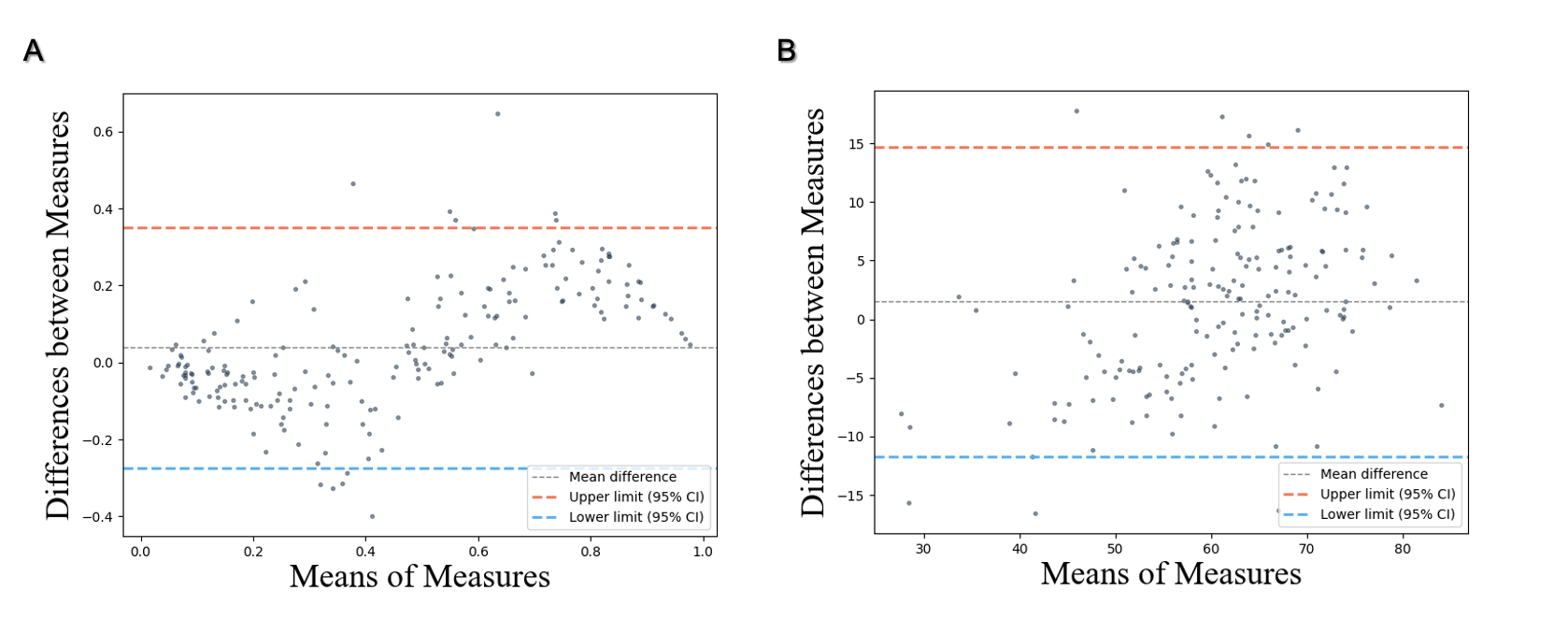


**Supplementary Figure 4. Bland-Altman analysis showing the differences between 3D T1 and 2D T1 images for (A) AD-specific atrophy similarity and (B) BAI.**

The orange dashed line represents the upper limit of the 95% CI, the blue dashed line represents the lower limit of the 95% CI, and the gray dashed line represents the mean difference.

Abbreviations: 3D, three-dimensional; 2D, two-dimensional; AD, Alzheimer’s disease; BAI, brain age index.

**Supplementary Table. Demographics of participants who have both the true 2D T1 and the 2D T1 from 3D T1 images**

|  | **Total** | **CU** | **DAT** |
| --- | --- | --- | --- |
|  | N = 364 | N = 170 | N = 194 |
| Age, years | 69.1 ± 9.7 | 64.8 ± 9.0 | 72.8 ± 8.7 |
| Female, *N* (%) | 237 (65.1) | 107 (62.9) | 130 (67.0) |
| Education, years | 9.8 ± 5.4 | 11.2 ± 4.9 | 8.6 ± 5.5 |

The values are expressed as mean ± standard deviation or number (percentage).

Abbreviations: CU, cognitively unimpaired; DAT, dementia of Alzheimer's type
